# Profile of the Users and the Most Visited Topics of a Pediatric eHealth Website

**DOI:** 10.3390/ijerph182111248

**Published:** 2021-10-26

**Authors:** Bruno José Nievas-Soriano, Gracia María Castro-Luna, Sonia García-Duarte, María del Carmen González-López, Tesifón Parrón-Carreño

**Affiliations:** 1 Department of Nursing, Physiotherapy and Medicine, University of Almeria, 04120 Almería, Spain; brunonievas@ual.es (B.J.N.-S.); tpc468@ual.es (T.P.-C.); 2 Obstetrics and Gynecology Unit, Hospital Torrecárdenas, 04009 Almería, Spain; sgarciaduarte@hotmail.com; 3 Primary Health Care District of Almería, Andalusian Health Service, 04006 Almería, Spain; mariac.gonzalez.lopez.sspa@juntadeandalucia.es

**Keywords:** eHealth, mHealth, telemedicine, pediatrics, users, profile

## Abstract

Parents need information about their children’s health, and the Internet has become an essential repository for this information. However, there is almost no information about which topics are the most searched, consulted, or shared, or about the main characteristics of pediatric website users. The main aim of this research was to describe the profile of the users of a pediatric website, which topics they searched for, which topics were the most consulted, and which were the most shared. Users’ demographic data were analyzed regarding their use of the Internet to search for information about health. A pediatric website for parents was analyzed. A 26-item questionnaire collected demographical features of the users of the website. Descriptive and analytical analyses were performed. Most participants used general search engines for their health searches, and the most searched keywords were prurigo, barking cough, and laryngitis. The most visited topics were unexplained fever, snots, and laryngitis. The most shared were snots, lipotomy, and dizziness. The users were mainly women (67.8%), with an average age of 38.8 years, and one or two children (89%) with a mean age of 4.6 years. The users who mainly used the Internet for health purposes were women of younger age, and with a higher educational level.

## 1. Introduction

The interest of users in the Internet as a tool for seeking health-related information is growing [1]. Parents need information about their children’s health, and the Internet is a growing resource from which to obtain this information [2]. For this reason, parents are increasingly accessing the web in search of information on health [3,4]. However, there are no references about the main characteristics of these users and how they may affect the use of health portals [5].

According to the literature, women use the Internet more than men in regard to health [3,6,7,8,9,10], and they also participate more in studies on eHealth interventions [11]. Young adults seem to perform more health searches on the Internet [3,7,8,9,12,13] due to their higher digital skills [14]. More educated users use the Internet more in regard to health [7,9,12,13,15,16], as do people with higher incomes [13,17,18]. Geographically, according to some authors, users who live in urban areas perform more health searches on the Internet [11], while others point out that this would be the case for those who live further away from medical services [15].

In the pediatric setting, the likelihood of performing health searches on the Internet could be related to the number of visits to a pediatrician [3,15]. These health searches occur even before a visit to a doctor [4]. Although little is known about how they are performed [19,20], there does seem to be a consensus in the literature that most parents use general search engines, such as Google, to perform searches on their children’s health [3,18,21,22,23,24,25,26]. Only 1% of the users use social media such as Facebook [3], although their use is increasing [14,23,27]. Parents of children with serious diseases are more active searchers [4,6], as are those of children with chronic diseases [6,16,28,29], although some authors have found no differences in the latter group [30]. However, there is no scientific literature on what parents are searching for when they arrive at a pediatric website, the topics they consult most on these websites, and how they share them.

Therefore, the main objective of this study was to describe the profile of the users of a pediatric website, written in an easy language and with a simple interface, and to find out what topics they searched for to access the website, what topics they consulted within the website, and what topics they shared with other users. In addition, the users’ demographic data were analyzed in regard to their use of the Internet to search for health topics.

## 2. Materials and Methods

For the study, we used a pediatric website for parents, written in Spanish, open access and free, where 338 pediatric topics with information based on scientific evidence about symptoms, diseases, and care of a healthy child, written in simple language by a pediatrician, could be located and consulted. The website was available at https://notodoespediatria.com (accessed on 2 October 2021), and it was developed using the free web hosting service WordPress.com (Automattic Inc., San Francisco, CA, USA). The main objective of the website was to allow parents to access evidence-based pediatric information, and it was certified by the Health on the Net Foundation. The interface of the website was designed to be easily usable by any kind of user. The background was white, the text black, and the images used were simple drawings with soft colors. The different sections of the website were accessible from a menu located at the head of the home page. On the right, a search engine allowed users to find any term within the website. When this research was performed, the website had been working for five years and six months.

Data on visits, visitors, most visited topics, search keywords, most shared topics, and shared topics were obtained from the WordPress servers. The two units of measurement for website traffic were visits and unique visitors: visits were counted when a visitor loaded a page, and unique visitors were counted when a user was detected for the first time in a specific period.

A 26-item questionnaire was designed to obtain a demographic profile of the website users (Table 1). No personal data were collected that would allow for the identification of participants. The questionnaire was developed using Google Forms (Google L.L.C., Mountain View, CA, USA) and remained active for website visitors for eight weeks. An informed consent form was displayed on the first page of the questionnaire, informing participants about the conditions of the study, its objectives, that the questionnaire was anonymous and therefore did not collect personal data, and that participants could leave the questionnaire at any time. No economic incentives were offered.

To perform univariate analysis, central tendencies and dispersion measurements were calculated for quantitative variables; absolute and relative frequencies were used for qualitative variables. The Kolmogorov–Smirnoff test was applied to establish the goodness of fit to normality for the variables studied, to determine the use of parametric or non-parametric tests. For means and proportions, 95% confidence intervals (CIs) were calculated. To perform bivariate analysis, the non-parametric Mann–Whitney U test was used for the contrast of the hypothesis of the equality of quantitative variables; for qualitative ones, the Pearson chi-squared test was used; and for the correlation of quantitative variables, Spearman’s rho was used. The statistical analyses were performed using S.P.S.S. version 26 (I.B.M. Inc., Armonk, NY, USA).

All the procedures described in this study were approved by the Human Research and Bioethics Committee of the University of Almería (Spain), reference number UALBIO2020/023.

## 3. Results

This section may be divided by subheadings. It should provide a concise and precise description of the experimental results, their interpretation, as well as the experimental conclusions that can be drawn.

### 3.1. Most Visited Topics, Most Used Keywords, and Most Shared Topics

At the time of data collection for this study, the website had been working over five years and six months, and it had received 2,909,785 visits, made by 2,432,167 unique visitors. The 25 most visited topics during are shown in Table 2. These 25 topics constituted 70.5% of the total number of visits (n = 2,052,102). The most visited topics were unexplained fever, snots, and laryngitis. The least visited topics, of the 338 posted on the website, were: alcohol intake in adolescents (n = 22), compulsive intake in children (n = 16), and suicide attempts in children and adolescents (n = 15).

WordPress detected 43,559 terms, words, and phrases users used in general search engines, such as Google, to access entries and pages of the website. The 25 most used keywords are shown in Table 3 and constituted 37.2% (n = 16,215) of the keywords used to access the website. The most frequently used were prurigo (used 3735 times), kennel cough (1848 times), and laryngitis (1296 times). Some search engines did not disclose search terms for privacy reasons, so they were shown as unknown search terms when search terms were not known. The total number of unknown search terms was 1,774,427.

Some of the topics of the website were shared, via the links available on the WordPress platform itself, a total of 2949 times. The most used social media were Facebook (1688 topics shared) and Twitter (1259 topics shared). The most shared topics are shown in Table 4. These include snots in children (shared 124 times), syncope, lipotomy and dizziness (shared 97 times), and laryngitis (shared 86 times).

### 3.2. Demographical Aspects of the Survey Participants

During the eight weeks that the questionnaire was available, the website received 117,032 visits from 98,577 unique visitors, of which 0.52% (n = 516) participated in the study by completing 516 valid questionnaires. Their main demographical features are shown in Table 5. The mean age of the participants of the study was 38.8 years, with a standard deviation (S.D.) of 6.1 years; 67.8% (n = 350) were women; 73.8% (n = 381) had university or higher education; 65.6% (n = 339) reported a household income of over 26,000 euros per year; 92.2% (n = 476) resided in Spain; and 78.1% (n = 403) lived in urban areas.

### 3.3. Demographic Aspects of Participants’ Children

The demographical features of their children are shown in Table 6. A total of 89% of the study participants (n = 459) had one or two children; 14.5% (n = 75) had children with chronic diseases; and 3.9% (n = 20) had children with serious diseases. Regarding the frequency with which they visited a pediatrician, 52.5% of the participants (n = 271) reported visiting a pediatrician three or fewer times a year; 47.4% of participants (n = 154) reported visiting a pediatrician between four and seven times a year; and 17.6% of participants (n = 91) reported visiting a pediatrician more than eight times a year.

### 3.4. Use of the Internet for Health Aspects

Regarding the use of the Internet for health aspects (Table 7), a total of 98.4% of the participants (n = 508) had access to the Internet on their smartphones, and 53.3% (n = 275) regularly accessed the Internet using that device. A total of 95.3% of the participants (n = 492) accessed the Internet several times a day, and 94.2% of the participants (n = 486) used the Internet for health searches. A total of 81.8% of the participants (n = 422) used general search engines for these searches; 72% of the participants (n = 371) relied on the Internet for health issues related to them; 65% (n = 335) relied on the Internet for health searches related to their children; and 86.1% of the participants (n = 444) reported that they could find information about children’s health on the Internet. A total of 50.4% of the participants (n = 260) reported that the information found on the Internet had never influenced their decision to visit a doctor, and 41.5% (n = 214) reported that it had influenced their decision to visit a pediatrician.

### 3.5. Use of the Analyzed Website

Regarding the data related to the specific use of the website (Table 8), a total of 45.5% of the participants (n = 235) knew about the website through social networks, and 54.7% of the participants (n = 282) accessed it using their smartphone. A total of 51.6% of the participants (n = 266) had known about it for more than a year, and 80.4% (n = 415) had accessed it between one and ten times. A total of 25.8% of the participants (n = 133) stated that they were searching for information, before visiting a pediatrician, when they accessed the website for the first time.

### 3.6. Bivariate Analysis

Bivariate analysis of the data stated these following aspects: A total of 96.6% of the women in our study (n = 338) reported using the Internet for health searches, compared to 89.2% (n = 148) of the men (chi-square = 11.30; *p* < 0.001). The mean age of participants who reported using the Internet for health searches (38.6 years) was significantly lower than the mean age of those who did not (41.8 years) (Mann–Whitney U; *p* = 0.003). A total of 95.5% of participants (n = 364) with an undergraduate or graduate degree reported using the Internet for health searches, compared to 88.4% of participants (n = 99) with a primary or secondary school education (chi-square = 7.73; *p* = 0.005). Among the study participants, no association was found between household income level and greater use of the Internet for health searches (chi-square value, likelihood ratio = 8.6; *p* = 0.070). No differences were found between the use of the Internet for health searches and whether the participants lived in urban or rural areas (chi-square value, likelihood ratio = 0.038; *p* = 0.084).

There was a positive correlation between participants’ trust in the Internet for consulting about their own health and for consulting about their children’s health (Spearman’s rho correlation = 0.844; *p* < 0.001). There was no difference between using the Internet for health searches and having a child with a chronic illness (chi-square value = 0.321; *p* = 0.570) or with a severe illness (chi-square value, continuity correction = 0.750; *p* = 0.380). There were no differences in the frequency with which they visited a pediatrician and income level (Spearman’s rho correlation −0.064; *p* = 0.144). No statistically significant differences were found between using the Internet to perform health searches and the number of times they visited a pediatrician (Mann–Whitney U; *p* = 0.111).

There was a positive and significant correlation between the degree to which the participants in the study considered themselves capable of finding information about children’s health on the Internet and their confidence to consult health issues about their children on the Internet (Spearman’s rho correlation = 0.309; *p* < 0.001). There was also a positive and significant correlation between the degree to which they considered themselves able to find information about children’s health on the Internet and how confident they felt when using websites similar to the one analyzed (chi-square value, likelihood ratio = 19.196; *p* = 0.014). Women with a higher education level reported feeling more able to find information about children’s health on the Internet than those with primary or secondary education (chi-square value, likelihood ratio = 9.11; *p* < 0.001).

## 4. Discussion

Users, and specifically parents, increasingly demand more reliable health resources on the Internet [1,2,3,4]. Nevertheless, it is not easy to develop these resources without clearly knowing the profile of the users that are going to use them. As there are few studies about this specific aspect, we decided to perform research to acknowledge the main features of users of such websites. This could help actual and future developers of eHealth websites, especially if they are focused in pediatrics. Therefore, the main objective of this study was to describe the profile of the users of a pediatric website and to find out what topics they searched for to access the website, what topics they consulted within the website, and what topics they shared with other users. The demographic users’ data were analyzed concerning their use of the Internet to search about health topics.

According to numerous authors, most parents who use the Internet as a source of health information are women [6,7,16,27,31], who also tend to participate more in studies related to the use of the Internet for health [11], findings that we have confirmed in our results. Highly educated women tend to have higher eHealth competencies [31,32], which we also found in our study. In terms of age, young users tend to be more likely to use the Internet for issues about health [7,10,12,13,18], as we have also found. However, according to other studies, most parents would be younger than 35 years old [6,9], but in our study the mean age of the participants was higher (38.8 years).

Users with a higher educational level use the Internet more to search for health information [7,9,12,13,15,16,18], especially in the pediatric field [16], and the results of our study are concordant with this fact. On the other hand, there seems to be a positive association between having health competencies and trusting the Internet as a source of information [33]. The results of our study also seem to go in this direction.

For some authors, people with higher incomes use the Internet more concerning health aspects [13,17,18], while others have found no differences in this regard [15,30]. The results of our study are more in agreement with these latter studies. On the other hand, users who live in cities make more health searches on the Internet [11], while others point out that this would happen in those who live further away from medical services [15]. However, there are no differences in this regard [3,34], as was the case in our study.

Some authors report that users search for health information as often for themselves as for their relatives [15], while other studies describe that users tend to use the Internet more for information about others [35]. Our results agree with the first statement, as our users used and relied on the Internet for their own health and for that of their children. The likelihood of performing health searches could be related to the number of visits to a pediatrician [3,15], although there are authors that have not found such an association [28], as is the case with our results. Users with lower income levels could also consult a pediatrician more often [28]. However, we found no differences in this regard among the participants in our study.

There are no references in regard to how parents search for information [19,20]. These searches occur even before going to the doctor [4], and most users use general search engines, especially Google [3,21,22,23,24,25,26], something with which our results agree. Our study found that the terms most frequently used in general search engines to reach the analyzed website were prurigo, barking cough, and laryngitis. However, the most consulted topics on the same website were those related to fever without cause, snots, and laryngitis. Only 1% of users seem to use networks such as Facebook [3] to search about health, a figure similar to our findings. The importance of social networks is increasing however [14,23,27], which is consistent with the fact that most of the participants in the study reported having found out about the analyzed website through this medium. Indeed, Facebook was the social media most frequently used to share topics published on the website. Therefore, social media could be a good starting point to attract more users to websites such as the one analyzed. An important aspect to consider here is the use of friendly interfaces.

Parents of children with serious illnesses are more active searchers [4,6]. The same is true of parents of children with chronic diseases [6,16,26,28,29], although some authors have found no differences in the latter group [30]. In our study, we found no differences between using the Internet to perform health searches and having a child with a chronic or severe disease, although this could be related to the generalist nature of the Web. Thus, and differently to other studies on this topic, our research collected some aspects that could be important for developers when researching future pediatric eHealth resources: we found no differences among users regarding incomes, or if they lived near or far from medical services. Our users relied on the Internet for their health and the health of their children, and we found no differences, among users, regarding the number of visits to a pediatrician.

These results highlight the importance of increasing the people involved with health searches and the use of reliable pediatric eHealth resources. In our opinion, more studies are necessary to evaluate different aspects of eHealth websites from the point of view of the users. This way, we could analyze aspects such as their usability or perceived utility, among others. This research could help developers to make attractive interfaces for all kind of users. For this research, we selected come variables that could be interesting from the point of view of researchers. Our analysis allowed us to define the profile of the users of a pediatric website, highlighting features that allow us to conclude that perhaps we need to develop more usable interfaces. This way, we could attract more general users, who are less involved with technology, to access reliable scientific eHealth websites. Future research could study the potential correlation among the variables selected in this research.

The main limitation of this work is that, although the participants participated randomly, they did it voluntarily, thus generating a selection bias, which is unavoidable in open, online surveys [36]. There are also some limitations in the statistics provided by WordPress: in regard to the terms that users used in general search engines, such as Google, to access the website analyzed, the number of unknown terms was considerable, which may lead to a bias when interpreting these data. Concerning the most shared topics, WordPress shows the number of those shared from their platform, but it is not possible to know the topics that were shared by direct copying of links. Finally, it is essential to consider that the data obtained in this study come from the analysis of a specific website. Albeit it had been functioning for more than five years, the external validity of the results and the conclusions should be considered with caution. The greatest strength of this study lies in the fact that the website had been functioning over five years and six months, and it had received 2,909,785 visits, made by 2,432,167 unique visitors, when this research was performed. Regarding the data collected through the questionnaire, the sample was 516 participants, a figure higher than that recommended by other authors for studies of this type [37,38]. Finally, it is important that the sample and the data obtained came from real users of a pediatric website, which provides considerable value to this study for potential considerations about future eHealth developments in pediatrics.

## 5. Conclusions

The main characteristics of the users of the analyzed website, about pediatrics for parents, were the following: users were predominantly female, with an average age of 38.8 years, most of them lived in urban areas, and had one or two children with a mean age of 4.6 years. Most accessed the Internet several times a day, mainly from their smartphones, conducted online health searches, considered themselves capable to find information about children’s health online, and relied on the Internet for health information about their children. The most visited topics were fever without cause, snots, and laryngitis. The most shared topics were snots, syncope, and laryngitis. Facebook was the social network where the topics of the analyzed website were shared the most.

The users who most frequently used the Internet to search about health were young women with a high level of education. No differences were found regarding income level, area of residence, whether they had children with chronic or severe diseases, or the frequency with which they visited a pediatrician. Although most of the participants in our study reported using general search engines to perform online health searches, prurigo, barking cough, and laryngitis being the most searched terms, most of them reported having found our website through social networks. Our results also highlight the importance of increasing the people involved with health searches and the use of reliable pediatric eHealth resources. This way, we could analyze aspects such as their usability or perceived utility, among others. These aspects could be helpful when designing an eHealth pediatric website for parents.

## Figures and Tables

**Table 1:** Demographical data collected.

**About User’s Profile**
Gender
Age
Level of study
Income level
Country of residence
Urban or rural world
**About Their Children**
Number of children
Age of their youngest child
Children with chronic illnesses
Children with severe illnesses
Number of pediatrician appointments within the last year
**Internet Use about Health**
The device usually used to access the Internet
If they had access to the Internet via their mobile phone
How often they accessed the Internet
If the Internet was used to perform searches about health
If the Internet had influenced a decision to go to the doctor
If the Internet had influenced a decision to go to a pediatrician
Frequency of health-related searches on the Internet
How much they relied on the Internet for their health
How much they relied on the Internet for their children’s health
If they felt capable of finding information about children’s health on the Internet
**About Access to the Pediatric Website**
How they knew about the website
How they accessed the website
How long they had known about the website
How many times they had consulted the website
What they were searching for the first time they accessed the website

**Table 2:** Twenty-five most visited topics of the website.

Twenty-Five Most Visited Topics of the Website	n	%
Fever for no apparent reason	330,466	10.4
Snots in children	283,590	9.8
Laryngitis (barking cough)	252,520	8.7
Presence of blood in stool (rectorrhagia)	181,319	6.2
Syncope, lightheadedness, dizziness, or fainting	146,912	5.1
Children who poop without meaning to (encopresis)	111,338	3.8
Swollen lymph nodes or lymph nodes	94,110	3.2
Prurigo (skin lesions or papules)	89,004	3.1
Cephalohematoma and caput succedaneum	48,270	1.7
Umbilical cord infection (omphalitis)	47,769	1.6
Dizziness and vertigo in children	42,321	1.5
Dental caries	41,613	1.4
Bleach or caustic poisoning	40,675	1.4
Parasitic infections in children	40,653	1.4
Infant colic	37,358	1.3
Facial paralysis in children	30,354	1.0
Large head (macrocephaly)	30,235	1.0
Fever	28,875	0.9
Constipation	27,664	0.9
Diarrhea or acute gastroenteritis	27,061	0.9
Aphthous ulcers (stomatitis, mouth sores, or ulcers)	25,913	0.8
Anemia of infancy (or physiological anemia of lactation)	24,333	0.8
Enlargement of the spleen (splenomegaly) in children	24,307	0.8
Normal psychomotor development in 6 to 11 years old children	22,962	0.8
Ibuprofen poisoning in children	22,480	0.7
Total	2,052,102	70.5

**Table 3:** Most used keywords in general search engines.

Most Used Keywords in General Search Engines	n	%
Prurigo	3735	8.6
Barking cough	1848	4.2
Laryngitis in children	1296	2.9
Fever in children with no apparent cause	1092	2.5
Macrocephaly	883	2.0
Cephalohematoma	849	1.9
Kennel cough in children	687	1.6
Fever without symptoms	663	1.5
Childhood laryngitis	463	1.1
Thelarche	438	1.0
Fever in children without symptoms	434	1.0
Laryngitis	421	0.9
Omphalitis	352	0.8
Fever without symptoms in children	328	0.7
Kennel cough in children	320	0.7
Fainting in children	305	0.7
Laryngitis in infants	262	0.6
Fever with no apparent cause	252	0.6
Bloody stools in children	240	0.6
Fever without apparent cause in children	239	0.6
Gibbering	228	0.5
Dizziness in children	226	0.5
Snotty throat, baby	224	0.5
Lipotomy in children	220	0.5
Pediatrics	210	0.5
Total	16,215	37.2

**Table 4:** Most shared topics from the website.

Most Shared Topics from the Website	n	%
Snots in children	124	4.2
Syncope, lightheadedness, dizziness, or fainting in children	97	3.3
Laryngitis (barking or hoarse cough)	86	2.9
Fever for no apparent reason	69	2.3
Infant colic	45	1.5
Limping in infancy	42	1.4
Children who poop without meaning to (encopresis)	41	1.4
Pharyngitis, tonsillitis, and pharyngotonsillitis	39	1.3
Prurigo (skin lesions or papules)	35	1.2
All about Bexsero, the vaccine against meningitis	32	1.1
Hallucinations in children (phobic hallucinations)	32	1.1
Swollen lymph nodes or lymph nodes	32	1.1
Food allergies in children	31	1.1
Inflammation of the gums (gingivitis)	30	1.0
Homepage	30	1.0
Large head (macrocephaly)	27	0.9
Presence of blood in stool (rectorrhagia)	25	0.8
Constipation	24	0.8
Aphthous ulcers (stomatitis or mouth ulcers)	23	0.8
Umbilical cord infection (omphalitis)	23	0.8
Sleep in children	22	0.8
Coffee-with-milk spots on the skin in children	20	0.7
Premature thelarche (breast development)	20	0.7
Children who do not eat properly	20	0,7
Bleach (caustic) poisoning	19	0.6
Total	2949	33.5

**Table 5:** Demographic data of participants.

**Distribution by Age**	**Mean**	**S.D.**
	38.8	6.1
**Distribution by Sex**	**n**	**%**
Female	350	67.8
Male	166	32.2
**Distribution by the Level of Study**	**n**	**%**
Masters or postgraduate degree	128	24.8
University studies	253	49.0
Secondary school or baccalaureate	92	17.8
Primary or school graduate	20	3.9
Others	23	4.5
**Distribution by Income Level**	**n**	**%**
More than EUR 75.000/year	57	11.0
Between EUR 51.000 and 75.000/year	92	17.8
Between EUR 26.000 and 50.000/year	190	36.8
Between EUR 11.000 and 25.000/year	133	25.8
Less than EUR 10.000/year	44	8.5
**Distribution by Place of Residence**	**n**	**%**
Spain	476	92.2
Central/South America	24	4.7
North America	12	2.3
Another European country	3	0.6
Africa	1	0.2
**Distribution by Geographical Area**	**n**	**%**
Urban	403	78.1
Rural	113	21.9
Total Number of Participants	516	100

**Table 6:** Demographic data of participants’ children.

**Distribution by Age**	**Mean**	**S.D.**
	4.6	4.0
**Distribution by the Number of Children**	**n**	**%**
One	182	35.3
Two	277	53.7
Three or more	39	7.6
None	18	3.5
**Children with Chronic Illnesses**	**n**	**%**
Yes	75	14.5
No	409	79.3
It is under study	32	6.2
**Children with Serious Illnesses**	**n**	**%**
Yes	20	3.9
No	490	95.0
It is under study	6	1.2
**Frequency of Pediatric Visits**	**n**	**%**
Between 0 and 1 time a year	125	24.2
Between 2 and 3 times a year	146	28.3
Between 4 and 7 times a year	154	29.8
Between 8 and 10 times a year	62	12.0
More than 10 times a year	29	5.6
Total Number of Participants	516	100

**Table 7:** Use of the Internet for health aspects.

**Device Usually Used to Access the Internet**	**N**	**%**
Smartphone	275	53.3
Computer	52	10.1
Tablet	20	3.9
Indistinct	169	32.8
**You Have Access to the Internet on Your Mobile Phone**	**N**	**%**
Yes	508	98.4
No	8	1.6
**The Frequency You Usually Access the Internet**	**N**	**%**
Several times a day	492	95.3
Once a day, or less	24	4.7
**Use of the Internet to Perform Health Searches**	**N**	**%**
Yes	486	94.2
No	30	5.8
**How Health Information Is Searched on the Internet**	**N**	**%**
Use of general search engines, such as Google	422	81.8
I access directly the websites I want to consult	84	16.3
Other ways (Facebook pages, online forums, etc.)	10	1.9
**Confidence on the Internet to Consult about Their Health**	**N**	**%**
A lot	7	1.4
Quite a lot	99	19.2
Something	265	51.4
Little	133	25.8
Nothing	12	2.3
**Confidence on the Internet to Consult about Their Children’s Health**	**N**	**%**
A lot	7	1.4
Quite a lot	80	15.5
Something	248	48.1
Little	150	29.1
Nothing	31	6.0
**The Extent to Which the Users Considered Themselves Capable to Find Information about Children’s Health on the Internet**	**N**	**%**
Highly skilled	63	12.2
Quite capable	199	38.6
Somewhat capable	182	35.3
Poorly trained	66	12.8
Not trained at all	6	1.2
**The Health Searches Have Influenced Whether to See a Doctor**	**N**	**%**
Yes	260	50.4
No	230	44.6
I do not know	26	5.0
**The Health Searches Have Influenced Whether to See a Pediatrician**	**N**	**%**
Yes	214	41.5
No	260	50.4
I do not know	42	8.1
Total Number of Participants	516	100

**Table 8:** Data related to the use of the analyzed website.

**How the Website Was Found**	**n**	**%**
Through search engines	61	11.8
Through social networks	235	45.5
Recommendation through a message	60	11.6
By verbal recommendation in a non-health environment	56	10.9
Verbal recommendation from a health professional	47	9.1
By two or more of these ways	45	8.7
None of these ways	12	2.3
**Which Device Was Used to Access the Website**	**n**	**%**
A smartphone	282	54.7
Computer	83	16.1
Tablet	21	4.1
Indistinct	130	25.2
**How Long the Website Was Known**	**n**	**%**
More than one year	266	51.6
Between 1 and 12 months	68	13.2
Less than a month	182	35.3
**How Many Times the Website Had Been Visited**	**n**	**%**
Between 1 and 5 times	288	55.8
Between 5 and 10 times	127	24.6
Between 10 and 20 times	61	11.8
More than 20 times	40	7.8
**What the User Was Searching for the First Time the Website Was Accessed**	**n**	**%**
Information before a pediatrician appointment	133	25.8
Expand on information given by a physician or pediatrician	109	21.1
Healthy child information	70	13.6
Information before going to the emergency room	53	10.3
Clarify something not understood in the consultation or the emergency room	44	8.5
Others	107	20.7
Total Number of Participants	516	100

## Data Availability

All data are contained within the article.
